# The expression of human leukocyte antigen by human ejaculated spermatozoa

**DOI:** 10.1002/mgg3.1005

**Published:** 2019-10-18

**Authors:** Nasrin Sereshki, Alireza Andalib, Ataollah Ghahiri, Ferdos Mehrabian, Roya Sherkat, Abbas Rezaei, David Wilkinson

**Affiliations:** ^1^ Department of Immunology School of Medicine Isfahan University of Medical Sciences Isfahan Iran; ^2^ Department of Obstetrics and Gynecology Al‐Zahra Hospital Isfahan University of Medical Sciences Isfahan Iran; ^3^ School of Geosciences University of Aberdeen Aberdeen Scotland UK

**Keywords:** HLA‐Ia, HLA‐II, spermatozoa

## Abstract

**Background:**

After coitus and insemination, an inflammatory response is evident in the female reproductive tract (FRT). Semen contains a variety of immune‐activating components that have a major role in the induction of an immune response in the FRT. One of the most important is (human leukocyte antigen) HLA molecules which are present in soluble form in seminal plasma and in membrane form on the surface of cells (such as epithelial and leukocytes) existing in semen. Nevertheless, there is considerable debate over the expression of HLA antigens by human spermatozoa. Considering the critical role of HLA molecules in reproduction and the induction of an immune response, it is very important to clearly define HLA expression by spermatozoa and the role of these molecules in sperm morphology, motility, and strength to fertilize an egg. Therefore, the objective of this study was to determine HLA expression by ejaculated spermatozoa. The results of this study will facilitate the design of future studies.

**Method:**

Semen samples were collected from 50 healthy men with normal semen status by masturbation after 2–3 days of sexual abstinence. After purification of normal spermatozoa, HLA class I & II expression was evaluated by quantitative real‐time PCR and flow cytometry methods.

**Results:**

The results showed the expression of both HLA class I & class II by spermatozoa. The results also showed that the expression of HLA class Ⅱ was significantly more than HLA class Ⅰ.

**Conclusion:**

Spermatozoa express both HLA class I & class II molecules.

## Graphical abstract

We deal with the unresolved question of whether human spermatozoa express HLA molecules or not. We clearly showed that HLA class I & II express by mature human spermatozoa.

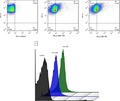

## 1 INTRODUCTION

After coitus and insemination, an inflammatory response is evident in the female reproductive tract (FRT). A variety of immune cells are recruited to the site of insemination. In this immunologic response, neutrophils first enter into the luminal space followed by other leukocytes such as macrophages, dendritic cells, and T cells (Bromfield, 2014; Robertson & Sharkey, 2016; Schjenken & Robertson, 2015). The consequence of this immune response is special immunologic homeostasis in the female reproductive tract which results in conditions suitable for tolerating semen and the semi‐allogeneic fetus and subsequently successful pregnancy (Schjenken & Robertson, 2014).

Semen contains a variety of immune activating factors which have a major role in the induction of an immune response in the FRT. One of these is HLA molecules which are present in soluble form in seminal plasma and also in membrane form on the surface of cells (such as epithelia and leukocytes) existing in semen (Bromfield, 2014; Katila, 2012; Robertson & Sharkey, 2016; Schjenken & Robertson, 2015; Schuberth et al., 2008).

There are two classes of HLA molecules; class I (HLA‐I) and class II (HLA‐II) (Ploegh, Orr, & Strominger, 1981). The HLA class I include HLA‐Ⅰa (HLA‐A, B, and C) and HLA‐Ⅰb (HLA‐G, E, and F) (Wei & Orr, 1990). HLA‐Ⅰa and HLA‐Ⅱ are *polymorphic HLA* and result in strong activation of the immune response (Ploegh et al., 1981). The HLA‐Ⅰa is expressed on the surface of almost all nucleated cells, while the expression of HLA class II antigens is limited to a few cells such as antigen‐presenting cells (APC), B cells, and activated T cells (Choo, 2007). These antigens have the main role in the immune response. In addition to immunological roles, these antigens play major roles in reproduction.

Many studies have been performed to show the critical roles of HLA molecules in reproduction, including: (a) Immune regulatory role of maternal and paternal HLA‐C allotype as well as HLA‐E, HLA‐G expressed by extravillous trophoblast (Kelly & Trowsdale, 2017; Moffett, Chazara, Colucci, & Johnson, 2016); (b) Presence of specific T lymphocytes directed to paternal HLA antigens in maternal lymphocyte repertoires in parous women (Toyoda et al., 2010); (c) The role of the maternal alloantibody against paternal HLAs in successful pregnancy (Khonina, Broitman, Shevela, Pasman, & Chernykh, 2013; Kishore et al., 1996; Orgad et al., 1999); (d) The association between recurrent spontaneous abortion (RSA) and increased HLA sharing among spouses (Beydoun & Saftlas, 2005; Meuleman et al., 2015); (e) The association between HLA variants with pregnancy complications such as RSA and preeclampsia (Akbari, Ahmadi, & Shahsavar, 2018; Colucci, 2017) and (f) Aberrant expression of HLAs at the feto‐maternal interface in preeclampsia (Tersigni et al., 2018).

Despite the key role of these molecules in reproduction, it has not yet been clearly defined the expression of polymorphic HLA molecules by spermatozoa. Spermatozoa is a major cell in reproduction. There is considerable debate over the expression of polymorphic HLA antigens by human spermatozoa. To the best of our knowledge, the last studies in this field were performed in 2000 and 2001 (Paradisi et al., 2000, 2001). In these studies, in addition to showing the expression of polymorphic HLA antigens by spermatozoa, it was proposed that some of these antigens may be involved in infertility.

It is probable that the variant of polymorphic HLA on the surface of spermatozoa may impact upon the activated immune response in the FRT (necessary for preparation of the FRT for embryo acceptance) during insemination. Also, it is possible that the variant of polymorphic HLA on spermatozoa may have some effects on spermatozoa morphology, motility and strength to fertilize an egg. Considering the key role of HLA molecules in reproduction and the association of these molecules with reproduction disorders, the understanding of polymorphic HLA expression by spermatozoa is considered very important. Therefore, the objective of this study was to determine polymorphic HLA expression (class Ⅰa and class Ⅱ) by ejaculated spermatozoa. The results of this study will facilitate the design of future studies.

## 2 MATERIALS AND METHODS

### 2.1 Ethical compliance

The protocol for this study was approved by the Ethics Committee of Isfahan University of Medical Sciences (Isfahan, Iran).

### 2.2 Subjects

Fifty healthy volunteers aged 23–50 years entered the study. The reproductive health status of the volunteers was confirmed by specialist doctors. Semen samples were collected by masturbation after 2–3 days of sexual abstinence. The samples were placed at room temperature for 60 min for liquefying. After liquefaction, semen quality was assessed according to WHO standard guidelines (WHO, 2010). Samples with normal quality (according to WHO reference intervals for values of semen parameters) were selected for the assessment of HLA expression. Informed consent was obtained from all subjects who participated in this study. Table 1 shows mean and standard deviation (*SD*) of subjects.

**Table 1:** Mean ± *SD* of semen parameters

Parameters	*n*	Mean	*SD*
Volume of semen (ml)	50	3.5	1.1
Liquefaction time (minute)	50	32.2	10.2
PH value	50	7.4	0.15
Concentration of spermatozoa × 10^6^/ml	50	70.5	24.2
Progressive mobility (%)	50	57.1	12.2
Overall mobility (%)	50	66.8	12.7
Non‐progressive mobility (%)	50	33.2	12.6
Immotile morphology of spermatozoa in %	50	2.8	1.3

Note

Leukocyte count in all samples was less than 1 milion/ml.*n*: number of sample

### 2.3 Sperm purification

AllGrad (LifeGlobal® Group, Canada) gradient technique was used for purification of spermatozoa. AllGrad effectively separates the spermatozoa from debris, seminal plasma, epithelial cells, leukocyte, bacteria, and immature and abnormal spermatozoa. Two milliliters of AllGrad Wash (LifeGlobal® Group, Canada) were added to the liquefied semen sample and centrifuged at 350 g for 10 min. The pellet was re‐suspended in 1 ml of AllGrad Wash. Then two gradient solutions of 95% and 45% were prepared from AllGrad 100%. In each tube were carefully layered; 1 ml of AllGrad 90% gradient, followed by 1 ml of AllGrad 45% gradient and then 1 ml of the spermatozoa suspension. The tubes were centrifuged 400 *g* for 18 min. The spermatozoa pellet at the bottom of the centrifuge tubes was washed and re‐suspended in AllGrad Wash. For checking AllGrad solution quality, the purified spermatozoa solutions (a total of 15 samples were selected in different purification runs) were assessed by microscopic visualization for lack of non‐spermatozoa cell contamination.

### 2.4 Sperm RNA Extraction, Cdna Synthesis, And Quantitative Polymerase Chain Reaction (Qpcr) analysis

Total RNA from each sample was extracted using the RNeasy plus universal Mini Kit (contains genomic DNA eliminator) according to manufacturer's protocols (Qiagen, USA). cDNA synthesis was performed according to the protocol from the QuantiNova Reverse Transcription kit (Qiagen, USA). This kit also contained genomic DNA eliminator. The quantitative polymerase chain reaction (qPCR) amplifications were performed according to the QuantiNova SYBR Green PCR (Qiagen, USA) in a reaction with a total volume of 20 µl. The primer pairs for cDNA amplification are shown in Table 2. The qPCR conditions included an initial activation step at 95°C for 2 min, two‐step cycling denaturation at 95°C for 5 s and combined annealing/extension at 60°C for 10 s. The number of cycles was 45. All tests were assayed in triplicate. For assurance that residual DNA contamination does not interfere in experiments, we assayed control experiments in which no reverse transcriptase was added prior to the qPCR step. All samples were normalized to RPLP2 expression. The 2^−ΔCT^ method was used to determine the relative expression levels of samples. Optimization and validation method of real‐time PCR assay is summarized in Table 3.

**Table 2:** Primer pairs for cDNA amplification

Gene	Primer	The size of the product	Length	tm	GC%
HLAI‐B	Forward primer: ACCAGACCAGCAGGAGATA Reverse primer: AGCTCCCTCCTTTTCCAC	262	19 18	56.97 56.08	52.63 55.66
HLA‐DRA	Forward: CCTGACCAATCAGGCGAGTT Reverse: GTTGGCCAATGCACCTTGAG	141	20 20	60.0 60.04	55 55
RPLP2	Forward: GAAGATCTTGGACAGCGTGG Reverse: ACCAGCAGGTACACTGGCA	133	20 19	58.64 61.15	55 57.89

**Table 3:** Optimization and validation of real time PCR assay

Negative control	Mouse PBMC was used as negative control during the set‐up stage. Mouse cells do not express human MHC class I & II (HLA class I & II)
Positive control	We used human PBMC as positive control during the set‐up stage
Primers	The characteristic of primers is summarized in Table 2. Primers were not exon junction. Therefore, we used RNA extraction and cDNA synthesis kit with genomic DNA eliminator (description in method section)
Choice of internal control for normalization	Three reference genes were tested including beta actin, GAPDH, and RPLP2. We selected RPLP2 because its expression was more stable than beta actin and GAPDH
Efficiency	To obtain suitable efficiency, we tested different concentrations of primer and cDNA. The concentration that had a suitable efficiency was selected for doing the test
Quantification strategy	The 2^−he^ method was used to determine the relative expression since the efficiency (E) of the target and reference genes was approximately equal. E _HLAI‐B_ = 0.996, E _HLAI‐DRA_ = 1.002, E _RPLP2_ = 0.994
Verification of amplification	This was checked by melt‐curve analysis and agarose gel electrophoresis
Primer dimer	This was checked by melt‐curve analysis, no template control (NTC) and the negative control (mouse PBMC)

### 2.5 RNA analysis on non‐denaturing agarose gel electrophoresis

The Agarose Gel Electrophoresis method was used to determine the quality of extracted RNA. For mammalian total RNA, two intensive bands at approximately 4.5 and 1.9 kb should be observed against a light smear. These bands represent 28S and 18S rRNA and they are absent in spermatozoa. Therefore, we used this method to confirm the purity of spermatozoa. Noteworthy, the absence of intact 28S and 18S spermatozoa rRNA is often used to confirm the absence of somatic contamination (Jodar et al., 2013).

Agarose gel (Sigma‐Aldrich, Germany) in the concentration of 1% and contained 2 µl green viewer (Pars tous biotechnology, Iran) was prepared. Five microliters of RNA extract and DNA ladder (Life Science Supplies, USA) mixed with loading dye were loaded in the gel. The gel was placed in the buffer tank and poured in 1X Tris/Acetate/EDTA (TAE) buffer. Running voltage and time were 90 V and 45 min, respectively. Then the agarose gel was placed on the UV transilluminator in the gel doc for imaging.

### 2.6 Flow Cytometry

The presence of HLA class I and II on the surface of spermatozoa was assessed by direct immunofluorescence using a BD FACSCalibur (BD Biosciences, USA) flow cytometer. Two tubes for each sample containing 1 × 10^6^ spermatozoa were prepared. One was incubated with phycoerythrin (PE) mouse anti‐human HLA‐ABC (clone: G46‐2.6, BD pharmingen, USA) and the other was incubated with phycoerythrin (PE) mouse anti‐human HLA‐DR (clone: G46‐6, BD pharmingen, USA) at room temperature for 30 min. After two washes with AllGrad Wash (400 *g* for 5 min), tubes were run through the flow cytometer. Data from at least 100,000 events were collected using forward scatter (a logarithmic amplifier) and side angle of light scatter (a logarithmic amplifier). As a negative control, we used unstained control. Isotype controls [Mouse IgG1, κ (clone: G46‐2.6, BD pharmingen, USA) and Mouse IgG2a, κ (clone: G46‐6, BD pharmingen, USA)] were used to determine background fluorescence (autofluorescence and non‐specific binding of antibodies). To remove background fluorescence, antibody titration was done and the optimal titer that showed minimum background was selected. Noteworthy, cell viability test was not performed because we removed abnormal and dead spermatozoa by AllGrad solution before staining. Fluorescence data were obtained with the logarithmic amplifier. We used flowJo vx software for data analyses.

## 3 STATISTICAL ANALYSES

Descriptive analysis of HLA class I & II expression in spermatozoa included mean and standard deviation (*SD*). We used the paired‐samples T‐test to analyze the significance of differences in expression of HLA class I & II by spermatozoa.

## 4 ANALYSIS OF RESULTS

**Figure 1:**
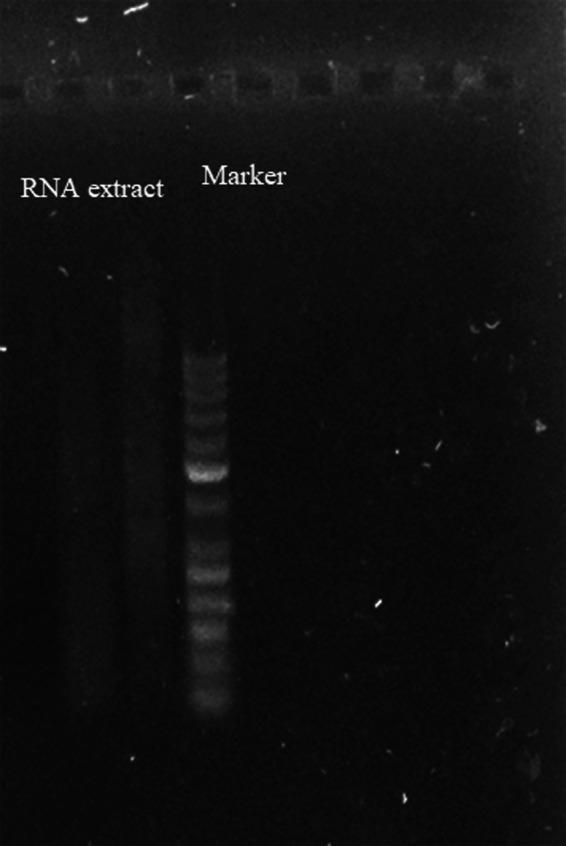
Image of agarose gel. Non‐denaturing agarose gel electrophoresis method was used to determine the quality of extracted RNA and purity of spermatozoa. The absence of bands representing 28S and 18S rRNA confirms no contamination with non‐sperm cells

The Agarose Gel Electrophoresis method was used to determine the quality of extracted RNA and purity of spermatozoa. Figure 1 shows the absence of bands representing 28S and 18S rRNA. This means that AllGrad solution could effectively separate spermatozoa from non‐spermatozoa cells.

**Figure 2:**
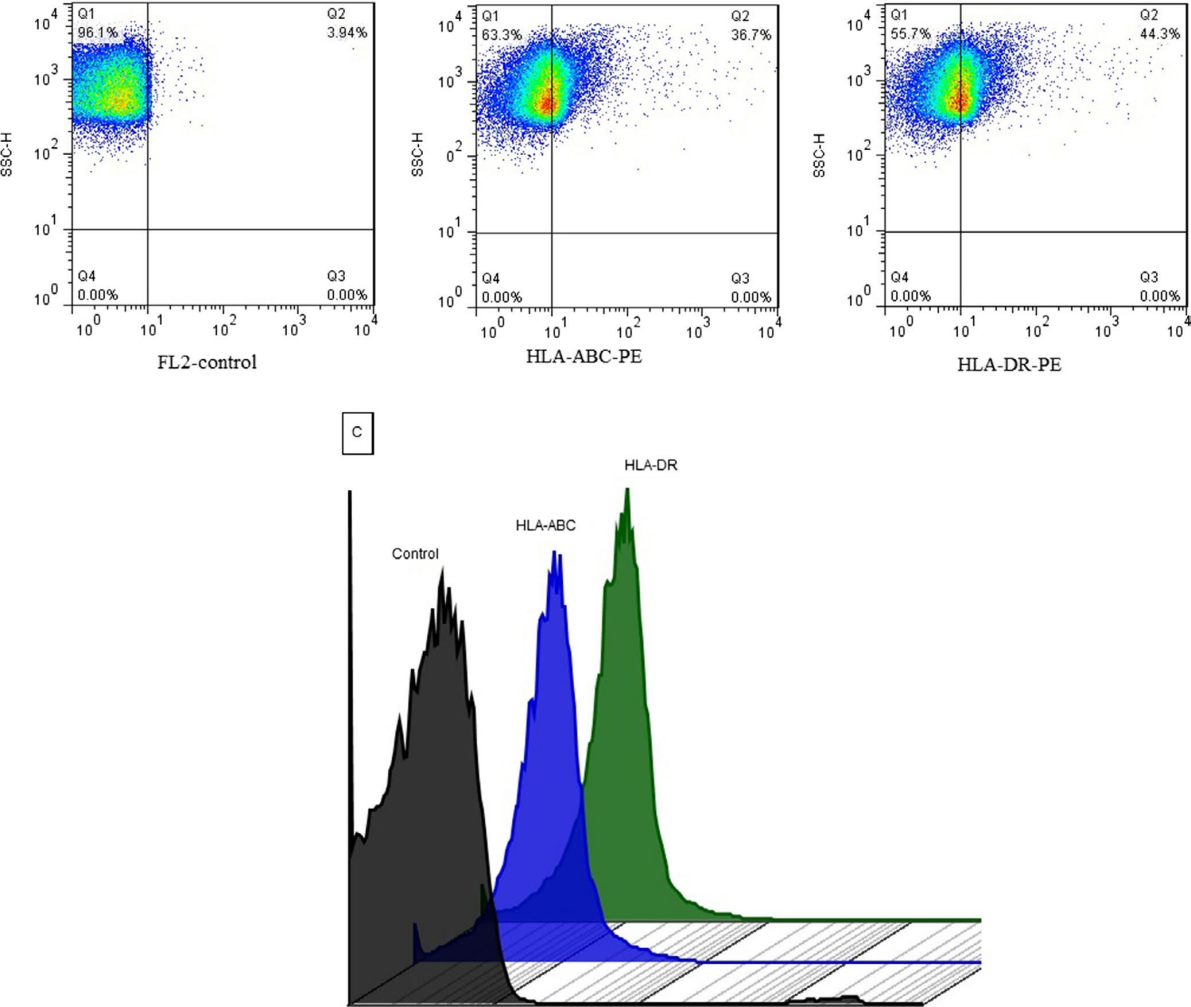
Representative flow cytometry dot plots and histogram of isotype control, unstained control, HLA‐DR, and HLA‐ABC. We used unstained control to differentiate the negative and positive population

Flow cytometric assay was used for evaluating HLA expression by spermatozoa. Results showed the expression of HLA class Ia & II on the surface of purified spermatozoa. In addition, results showed that the mean percentage of HLA class Ⅱ was significantly more than HLA class Ⅰa (Table 4; Figure 2).

**Table 4:** Flow cytometric and qPCR result

		Mean	*SD*		*p*‐value	
% of spermatozoa expressed HLA class I&II	HLA class I%	12.9	6.9		.009	
	HLA class II%	14.2	8.5			
Quantity of HLA protein expression (MFI)	HLA class I	4.82	1.46		.002	
	HLA class II	5.06	1.67			
The relative expression of HLA transcript	HLA class I	0.91	0.31		.000	
	HLA class II	1.03	0.33			

Note

*p* ≤ .05 considered significant. Paired sample T test showed that HLA class II expression is more than HLA class I.

Abbreviations: MFI, mean fluorescence intensity; *SD*, standard deviation

Transcript levels of HLA molecules were quantified with SYBR Green PCR. HLA mRNA expression levels were normalized to RPLP2. The data are shown in Table 4.

## 5 DISCUSSION AND RESULTS

In this study, we deal with the unresolved question of whether human spermatozoa express HLA molecules or not. By quantitative real‐time PCR or qPCR and flow cytometry methods, we clearly showed that HLA class Ia & class II is expressed by mature human spermatozoa. Also, we found that the expression by spermatozoa of HLA class Ia is less than HLA class II.

The expression of polymorphic HLA antigens by ejaculated spermatozoa has been a matter of controversy. Some studies reported that ejaculated spermatozoa express both HLA class Ia & class II (Chiang, Steuerwald, Lambert, Main, & Steinleitner, 1994; Martin‐Villa, Longás, & Arnáiz‐Villena, 1999; MartinVilla et al., 1996; Paradisi et al., 2000, 2001; Rodriguezcordoba, Regueiro, & Villena, 1986), but others described the contrary (Castilla et al., 1993; Haas & Nahhas, 1986; Law & Bodmer, 1978; Schaller, Glander, Ladusch, Westhoff, & Grossewilde, 1993). Also, some authors reported that spermatozoa express one of the HLA class Ia or class II (Bishara et al., 1987; Ohashi, Saji, Kato, Wakimoto, & Tanizawa, 1990). Researchers give some justifications for these conflicting data that include: (a) the use of polyvalent antisera in some of the early studies, which may be against not related HLA antigens; (b) lack of purity of sperm samples and contamination with other cells in semen; (c) the use of monoclonal antibodies with low specificity and/or affinity for detecting low levels of expression; (d) the presence of glycosidic residues in the sperm surface that may interfere with antibody‐antigen interaction; (e) cyclic expression of HLA by sperm that is controlled by hormone concentration; and (f) low sensitivity of some techniques. In this study, we tried to rule out some of these problems.

For purifying spermatozoa from other cells in semen, we utilized AllGrad solution. The principal of this method is based on discontinuous density gradient centrifugation. AllGrad solution effectively separates the spermatozoa from other cells and also immature and abnormal spermatozoa existing in seminal plasma. Therefore, it is improbable that our HLA positive results have been achieved due to contamination with HLA positive cells existing in semen, such as leukocytes and epithelial cells.

Previously used techniques for detecting polymorphic HLA expression by sperm have been micro‐immobilization tests (Law & Bodmer, 1978), micro‐agglutination cytotoxicity tests (Law & Bodmer, 1978), fluorescent‐labeling techniques (Haas & Nahhas, 1986; Paradisi et al., 2000, 2001), enzyme‐linked immunosorbent assay (ELISA) (Bishara et al., 1987), cellular binding radioimmunoassay (CB‐RIA) (Ohashi et al., 1990), and reverse transcription polymerase chain reaction (RT‐PCR) (Paradisi et al., 2000, 2001). As far as we know, our study was the first which applied qPCR for detection of HLA transcripts in spermatozoa. Between these techniques, qPCR and RT‐PCR are the most sensitive methods for the detection of mRNAs.

In this study, we detected mRNA of HLA class I & II molecules in spermatozoa by qPCR. To the best our knowledge, the studies which used the RT‐PCR method for detecting HLA transcripts in spermatozoa reported positive results. The studies which applied the flow cytometry method for the assessment of HLA expression by spermatozoa reported contradictory results. Paradisi et al. by indirect fluorescence immunoassay‐flow cytometry, showed the presence of HLA class Ia & II at the spermatozoa cell surface, contrary to Gillbert et al. who detected no HLA proteins. In both studies, the antibody was conjugated with FITC. It is known that the fluorescence intensity of FITC is low compared with PE, so fluorochrome is not suitable or detecting markers with low expression. Considering the possibility of the weak expression of HLA molecules by spermatozoa, we used monoclonal HLA antibodies conjugated with PE and consequently we were able to show the expression of HLA proteins by spermatozoa.

In a study in 2001, in addition to showing HLA class II expression by spermatozoa, the level of HLA class II expression was shown to be different in fertile and infertile men and a possible correlation of HLA class II expression with infertility was suggested. As mentioned above, we showed that the expression of HLA class II is more than that of HLA class Ia. The question arises, what is the role of HLA class II on spermatozoa? Noteworthy, HLA class II expresses on a few immune cells. It seems that the expression of HLA class II and HLA class Ia is an activating mechanism for the FRT immune system. As mentioned previously, the activation of the FRT immune system is indispensable for the preparation of the uterus for the acceptance of the embryo. We think HLA molecules at spermatozoa may have some roles in the occurrence of pregnancy and probably a change in the expression of these molecules or some of the variants of these molecules can result in infertility and pregnancy aberration such as RSA and pre‐eclampsia. Purposefully we will continue our study in determining spermatozoa HLA expression roles in pregnancy and its abnormality.

Based on the results in this study, it can be concluded that qPCR is a sensitive method for the detection of HLA transcripts in spermatozoa. Regarding methods applied in previous studies, flow cytometry (applying bright fluorochrome such as PE) is considered the best for detecting HLA proteins on spermatozoa. By the use of these methods, we clearly showed that spermatozoa express both HLA class Ia & class II. In our future research, we intend to concentrate on the role of HLA molecules in spermatozoa function and immune activation.

## CONFLICTS OF INTEREST

None declared.
