# Supplementary material for: The expression of human leukocyte antigen by human ejaculated spermatozoa
Source: Mol Genet Genomic Med. 2019 Oct 18;7(12):e1005. doi: 10.1002/mgg3.1005 (PMC6900355; doi:10.1002/mgg3.1005)
Supplement: Supplementary file 1 [file MGG3-7-e1005-s001.docx]

Table 1: flow cytometry result of each sample

| sample | HLA-I% | MFI of HLA-I | HLA-II% | MFI of HLA-II |
| --- | --- | --- | --- | --- |
|  | 19.8 | 5.93 | 22.3 | 6.02 |
|  | 8.74 | 3.2 | 10.4 | 3.3 |
|  | 7.61 | 3.12 | 9.51 | 3.28 |
|  | 21.3 | 4.3 | 23.6 | 4.47 |
|  | 8.68 | 2.92 | 11.19 | 2.96 |
|  | 9.65 | 2.98 | 9.9 | 3.02 |
|  | 12.5 | 3.49 | 15.2 | 3.32 |
|  | 10.2 | 3.19 | 19.81 | 3.12 |
|  | 10.5 | 3.19 | 10.55 | 3.08 |
|  | 18.7 | 5.74 | 14.2 | 5.13 |
|  | 18.8 | 5.7 | 14 | 5.13 |
|  | 11 | 4.52 | 10.4 | 4.3 |
|  | 9.65 | 4.18 | 9.9 | 4.2 |
|  | 20 | 5.52 | 20.3 | 6.11 |
|  | 9.74 | 3.71 | 10.3 | 3.76 |
|  | 6.14 | 4.7 | 9.47 | 5.21 |
|  | 5.73 | 4.28 | 7.25 | 4.68 |
|  | 13.7 | 3.96 | 5.53 | 4.35 |
|  | 11.73 | 3.58 | 4.26 | 4.15 |
|  | 4.25 | 3.51 | 7.65 | 4.03 |
|  | 7.97 | 3.99 | 8.99 | 4.09 |
|  | 14.46 | 4.79 | 15.3 | 4.88 |
|  | 13.63 | 4.68 | 15.6 | 4.88 |
|  | 5.69 | 4.43 | 5.6 | 4.63 |
|  | 13.4 | 5.8 | 12.9 | 5.99 |
|  | 12.3 | 5.14 | 14.7 | 6.86 |
|  | 13.5 | 5.83 | 13.8 | 5.92 |
|  | 13.7 | 5.7 | 13.9 | 6.07 |
|  | 13.2 | 5.75 | 14.2 | 6.14 |
|  | 11.2 | 4.79 | 11.5 | 4.81 |
|  | 19 | 7.32 | 26.4 | 8.18 |
|  | 16.4 | 8.18 | 32.1 | 8.67 |
|  | 27.4 | 7.73 | 36.5 | 8.81 |
|  | 20.1 | 8.09 | 36.5 | 8.81 |
|  | 10.8 | 5.93 | 12.3 | 10.02 |
|  | 12.74 | 3.23 | 10.4 | 6.3 |
|  | 12.61 | 3.1 | 15.51 | 10.28 |
|  | 21.3 | 4.3 | 23.6 | 4.47 |
|  | 16.68 | 2.92 | 12.19 | 12.96 |
|  | 10.65 | 3.98 | 10.9 | 3.02 |
|  | 12.5 | 4.49 | 12.2 | 6.32 |
|  | 10.3 | 4.19 | 9.81 | 3.12 |
|  | 10.4 | 3.99 | 9.55 | 4.08 |
|  | 18.1 | 5.74 | 16.2 | 5.13 |
|  | 10.8 | 3.54 | 12 | 5.13 |
|  | 11 | 4.52 | 10.46 | 4.3 |
|  | 9.95 | 4.18 | 9.84 | 4.2 |
|  | 14 | 5.52 | 20.3 | 6.11 |
|  | 9.14 | 7.91 | 9.5 | 7.76 |
|  | 12.14 | 4.7 | 11.47 | 5.21 |
